# Case report: Irinotecan-induced interstitial lung disease in an advanced colorectal cancer patient resurfacing decades after allogeneic bone marrow transplantation for aplastic anemia; a case report and narrative review of literature

**DOI:** 10.3389/fonc.2023.1215789

**Published:** 2023-06-16

**Authors:** Keisuke Baba, Yasuo Matsubara, Yoshihiro Hirata, Yasunori Ota, Satoshi Takahashi, Narikazu Boku

**Affiliations:** ^1^ Department of Oncology and General Medicine, IMSUT Hospital, Institute of Medical Science, The University of Tokyo, Tokyo, Japan; ^2^ Department of Diagnostic Pathology, IMSUT Hospital, Institute of Medical Science, The University of Tokyo, Tokyo, Japan; ^3^ Department of Hematology/Oncology, IMSUT Hospital, Institute of Medical Science, The University of Tokyo, Tokyo, Japan

**Keywords:** drug-induced interstitial lung disease (DILD), bone marrow transplantation (BMT), irinotecan, colorectal cancer, graft-versus-host disease (GVHD), late-onset non-infectious pulmonary complication (LONIPC)

## Abstract

Two mechanisms of drug-induced interstitial lung disease (DILD) have been reported: 1) direct injury of lung epithelial cells and/or endothelial cells in lung capillaries by the drug and/or its metabolites and 2) hypersensitivity reactions. In both mechanisms, immune reactions such as cytokine and T cell activation are involved in DILD. While past and present lung diseases and accumulative lung damage due to smoking and radiation are risk factors for DILD, the association between the immune status of the host and DILD is not well known. Herein, we report a case of advanced colorectal cancer with a history of allogeneic bone marrow transplantation for aplastic anemia more than 30 years prior, in which DILD occurred early after irinotecan-containing chemotherapy. Bone marrow transplantation might be a potential risk factor for DILD.

## 1 Introduction

Bone marrow transplantation (BMT) is an important treatment option for patients with hematologic malignancies and severe hematopoietic and immune system disorders (1, 2). While long-term survival can be obtained after BMT, recipients are also reported to be at an increased risk of secondary cancers (3–5). As the probability of secondary cancer increases with prolonged survival after BMT, the number of BMT recipients receiving chemotherapy for secondary cancers has recently been increasing (6–8). However, there are few reports on the efficacy and side effects of chemotherapy for secondary cancers in recipients of BMT. Here, we report a case of incurable interstitial lung disease induced by irinotecan-containing chemotherapy for advanced colorectal cancer more than 30 years after allogeneic BMT.

## 2 Case description

**Figure 1:**
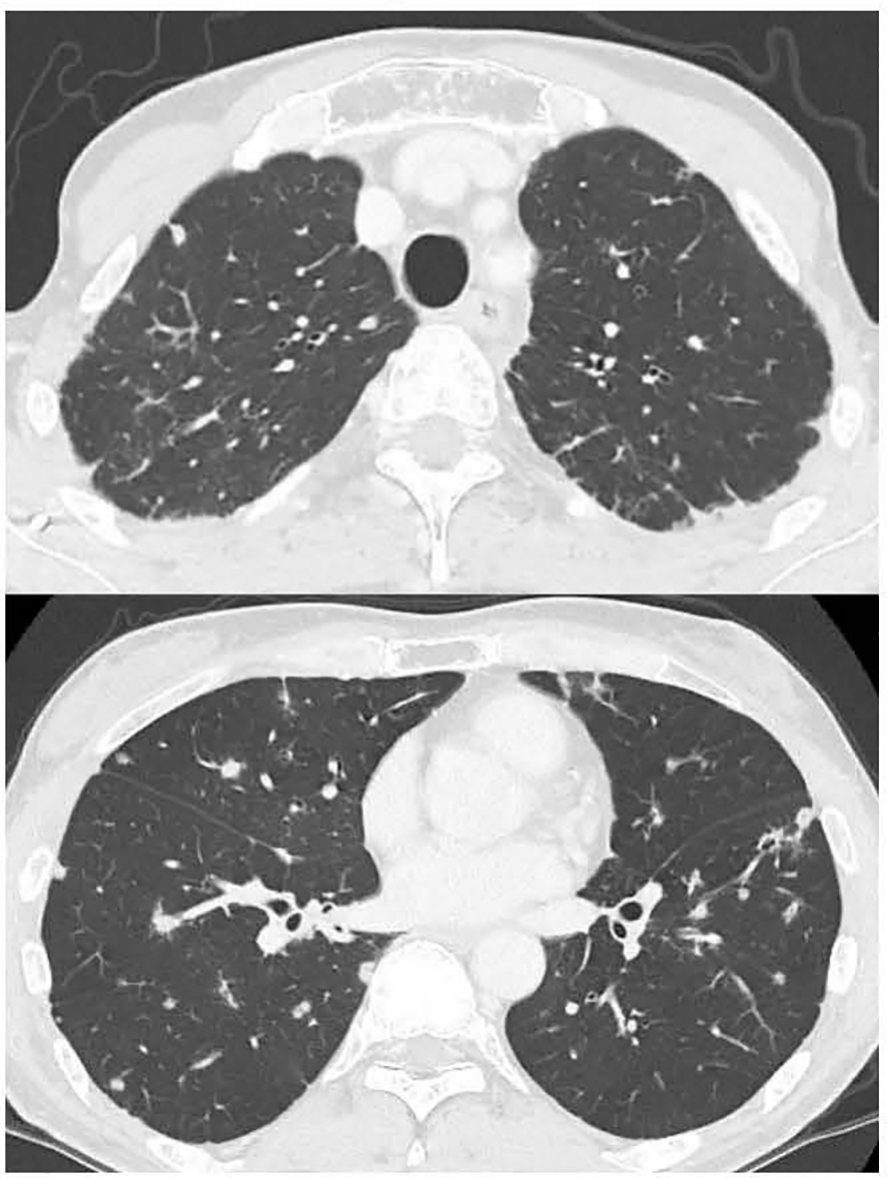
Chest CT showing multiple lung involvements at initial diagnosis.

**Figure 2:**
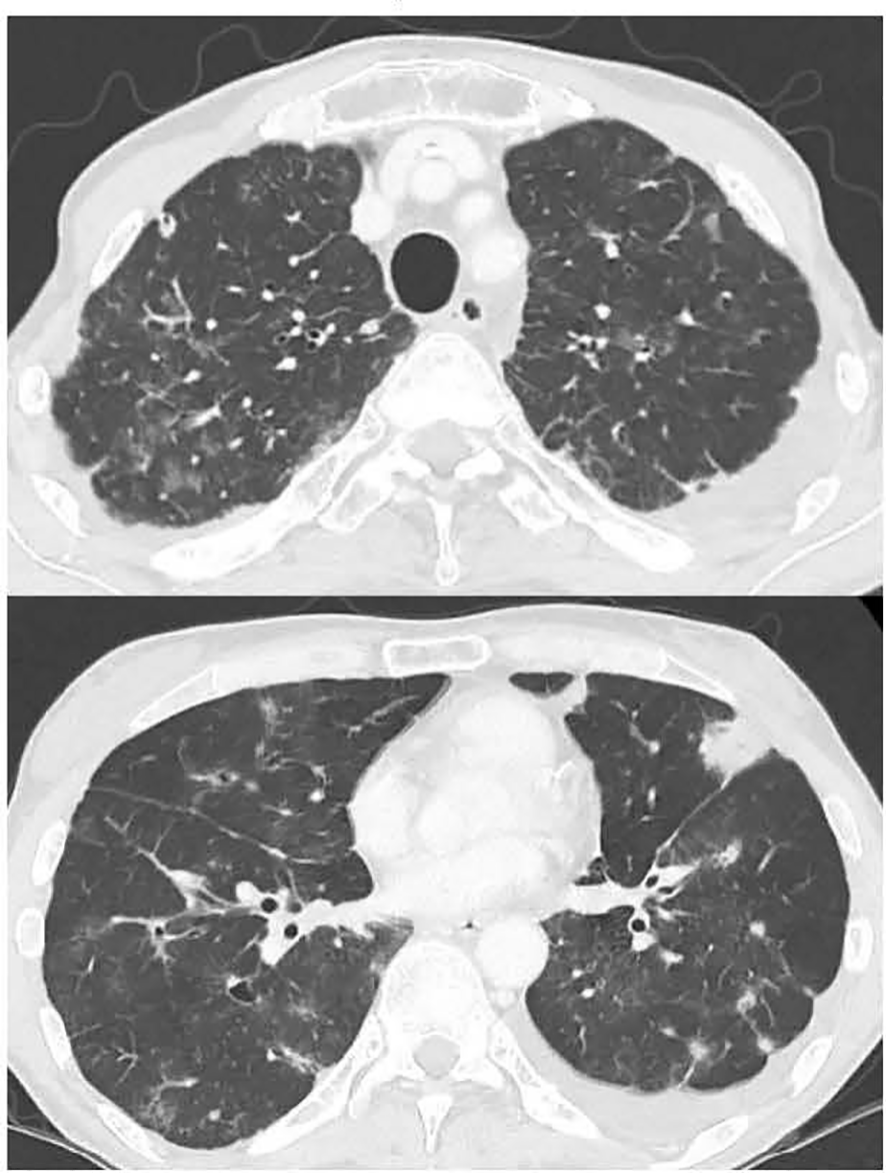
Chest CT showing the bilateral lung interstitial infiltrations.

**Figure 3:**
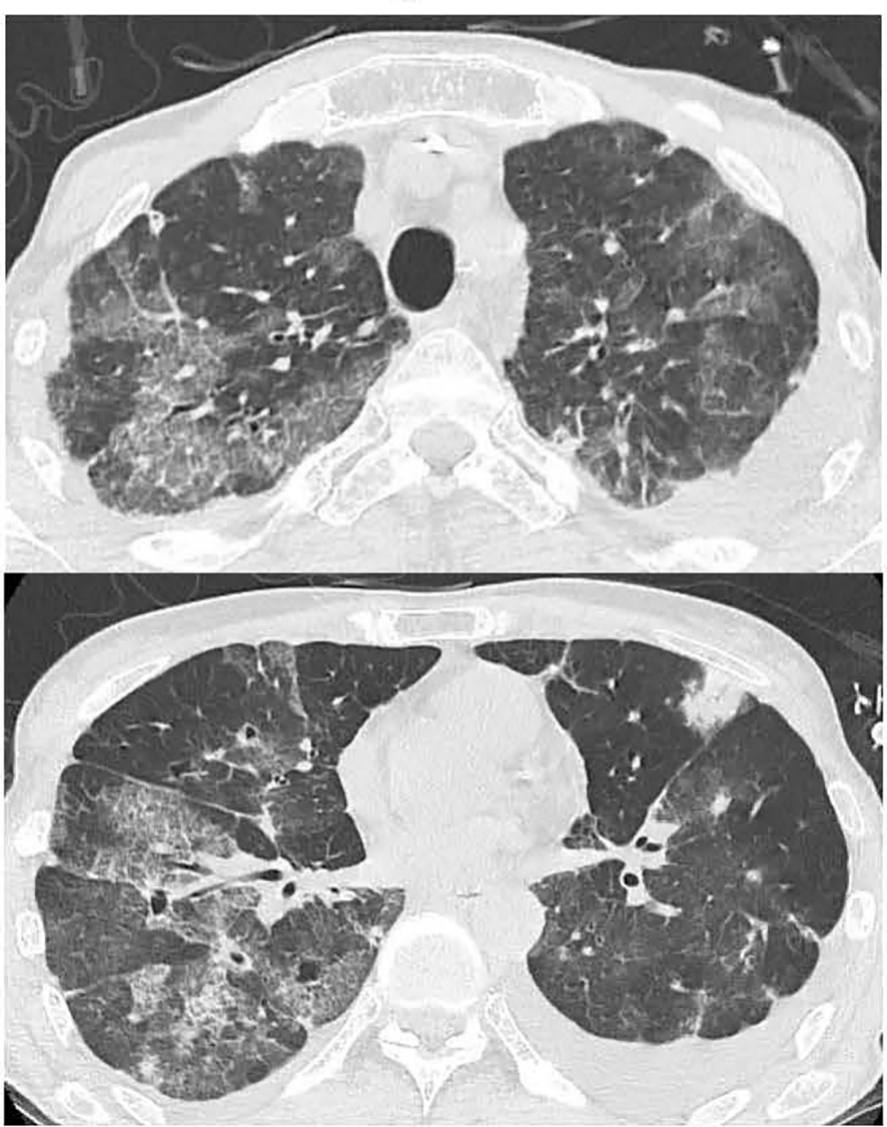
Chest CT showing exacerbation of bilateral lung interstitial infiltrate shadows on day 6 after admission.

**Figure 4:**
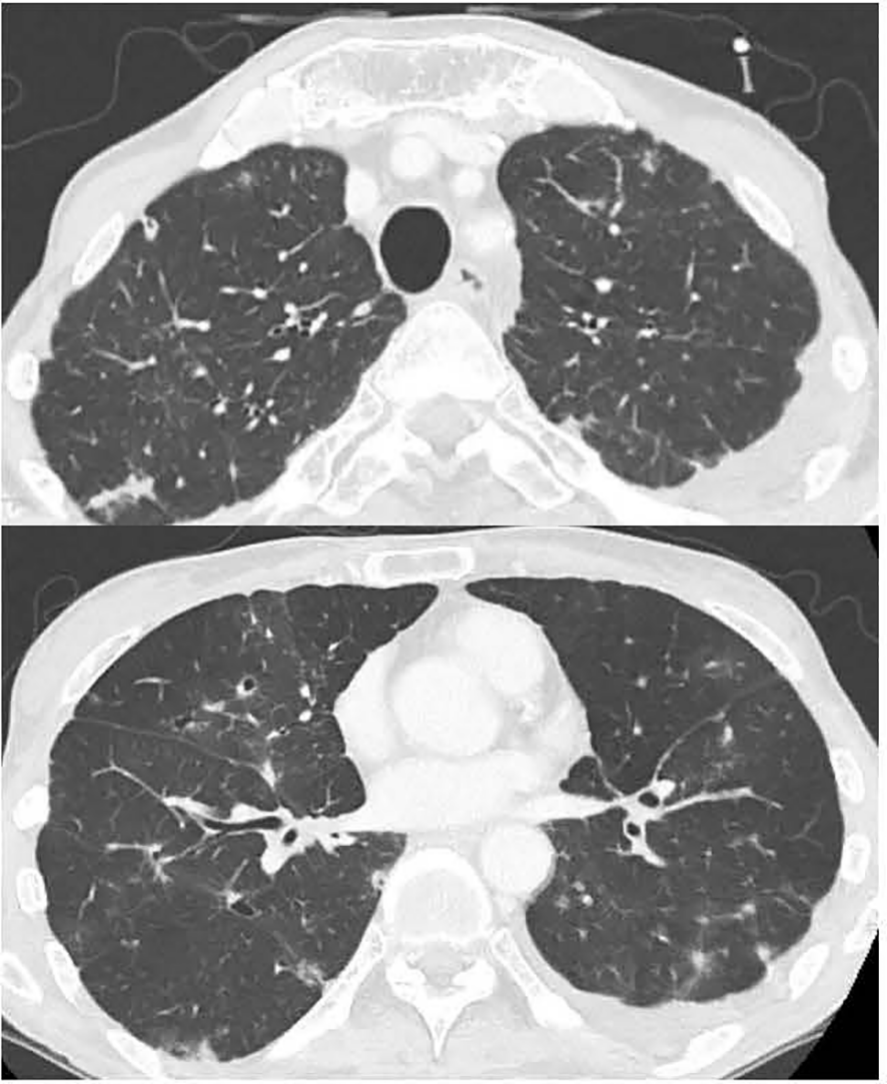
Chest CT showing the improvement in bilateral lung interstitial infiltrate shadows on day 21 of steroid treatment.

A 52-year-old male was diagnosed with advanced colorectal cancer, with multiple metastases to the lungs, liver, and peritoneum. Histology revealed well-to-moderately differentiated adenocarcinoma, in which the *KRAS* and *NRAS* mutation tests were negative and microsatellite instability was stable, but the *BRAF* ^V600E^ mutation was positive. In his medical history, he had received an allogeneic BMT from his elder sister for severe aplastic anemia 33 years ago. As preparative therapy prior to BMT, the patient received total lymph node irradiation (7.5 Gy) and cyclosporine at 50 mg/kg for four days. Only the bilateral lung apices were involved in the irradiated field, but no pulmonary changes suggestive of radiation pneumonitis were noted thereafter. He experienced a grade 1 skin rash as an acute graft-versus-host disease (GVHD) symptom after BMT, which disappeared clinically without treatment; however, a skin biopsy performed several months later showed pathological findings suspicious of chronic GVHD. During follow-up, there was no worsening of GVHD at the skin or other sites, including interstitial pneumonia, and the patient received no immunosuppressive agents at the time of diagnosis of colon cancer. He had no history of smoking or known allergies. The patient had received two doses of COVID-19 vaccine manufactured by Moderna 9 and 10 months prior to the diagnosis of colorectal cancer and polymerase chain reaction (PCR) test for COVID-19 was negative multiple times during the DILD treatment. The vaccination history other than COVID-19 was unknown. He had type 2 diabetes and used long-acting insulin. Because the sigmoid colon tumor invaded the left ureter and lower gastrointestinal endoscope could not pass through the primary site, the patient underwent transverse colostomy for colon obstruction due to the tumor. Before receiving chemotherapy, the patient experienced a severe cough due to lung metastasis but had no respiratory distress, with a pulse oxygen saturation (SpO2) of 95% in room air. Chest computed tomography (CT) showed multiple pulmonary nodules, but no abnormal findings suggesting accumulative lung damage such as fibrosis (**Figure 1**). All screening tests including blood cultures for bacterial infections and serological examinations for viral infections were negative. One month after surgery, 1st-line chemotherapy with FOLFIRI (5-fluorouracil [FU] bolus 400 mg/m^2^ + leucovorin 400 mg/m^2^ + irinotecan 150 mg/m^2^ followed by 5-FU 2400 mg/m^2^ in a 46-hour infusion) + bevacizumab (5 mg/kg) with palonosetron hydrochloride and dexamethasone as prophylactic anti-emetic therapy was started. He developed a fever without myelosuppression on day 6; however, the bacterial culture was negative, and there was no new lung infiltration on chest radiography. Naproxen (600 mg/day) was administered for the noninfectious fever, which quickly resolved. He was discharged from the hospital in a good general condition and received a second course of chemotherapy at our outpatient clinic with no severe adverse events. On day 15 of the second course of chemotherapy, the patient had no dyspnea. However, because his SpO2 was 92% in room air, the patient was admitted for oxygen demand. A CT scan showed no apparent tumor growth, but there were slight bilateral ground-glass shadows (**Figure 2**). A bronchoscopy was performed on the same day. There were no abnormalities in the endotracheal lumen such as apparent redness, edema, or neoplastic lesions; bronchoalveolar lavage (BAL) was performed in the middle lobe, and a biopsy of the peripheral lung was performed in the lower lobe of the right lung. The pathology showed that there was only fibrosis around the tumor as a tumor environment, and there was no obvious fibrosis in the background lungs consistent with the CT findings (**Supplementary Figure 1**). Bacterial and fungal cultures and PCR tests for *pneumocystis jirovecii* DNA and cytomegalovirus (CMV) DNA of the BAL fluid were negative. Pathology of the lung biopsy showed pulmonary metastasis of the colon cancer and no apparent findings of inflammation. Serum lactate dehydrogenase (LDH) and KL-6 levels were within the normal ranges, and the βD-glucan test was negative. The white blood cell count was 2,740/μL, neutrophil count 1,140/μL, and lymphocyte count 880/μL. The interferon-gamma release assay for tuberculosis was negative. Differential diagnoses were interstitial pneumonitis or atypical pneumonia. The clinical course after onset of DILD is summarized in the **Supplementary Figure 2**. There were no apparent clinical manifestations of chronic GVHD around the time of DILD. As his respiratory status was stable, the antimicrobial agent levofloxacin was initiated. Four days after admission, the patient’s respiratory status was stable. However, at night on day 5, the cough and dyspnea worsened and the oxygen demand increased. CT examination revealed expanded bilateral interstitial infiltrations (**Figure 3**). The white blood cell count was 6,260/μL, neutrophil count 5,440/μL, and lymphocyte count 720/μL, and the KL-6 level was mildly elevated (531 U/ml). On day 6, methylprednisolone (1 g/day) and piperacillin-tazobactam (4.5 g q6hr) were administered. Thereafter, the patient’s respiratory condition improved rapidly, and the shadows in the bilateral lung fields disappeared on day 21 after starting steroid therapy (**Figure 4**). The steroid dose was gradually decreased and steroid administration was switched to oral prednisolone 30 mg/day after day 22. The prednisolone dose was reduced to 20 mg/day on day 33 after starting steroid treatment, and the patient was scheduled to be discharged. Immediately before discharge on day 39, the patient complained of abdominal pain. Abdominal CT revealed a retroperitoneal hematoma and pseudoaneurysm in the anterior superior pancreatic duodenal artery. Because bleeding from the metastasis near the pancreatic head was suspected, the patient was transferred to a tertiary hospital, where intra-arterial embolization was performed. During the intervention, oral prednisolone was maintained at the same dose, and the patient returned to our hospital immediately after the intervention. On day 43, the lung infiltration reappeared. On day 44, steroid pulse therapy with methylprednisolone (500 mg/day) and piperacillin-tazobactam (4.5 g q6hr) was resumed. The methylprednisolone dose was tapered to 125 mg/day on day 47. On day 49, nasal high-flow therapy was initiated because oxygen demand increased. On the same day, intravenous cyclosporine (150 mg/day) was started after consultation with a hematologist, and methylprednisolone (500 mg/day) was retried. On day 51, laboratory tests reported the levels of aspartate aminotransferase (32 U/L), alanine transaminase (53 U/L), alkaline phosphatase (351 U/L), gamma-glutamyl transpeptidase (236 U/L), and total bilirubin (3.0 mg/dL). Considering the possibility of exacerbation of sepsis, an antifungal agent, micafungin (100 mg/day), was administered to treat all infectious diseases. On day 53, because serum CMV antigenemia tested on day 51 was positive (Cytomegalovirus antigen positive cell count was 200-206 positive cells per two slides, CMV IgG was > 250 AU/mL, CMV IgM INDEX was 1.04, absolute lymphocyte count was 280/μL), ganciclovir (500 mg/day) was added in combination with other antibiotics. The measurements of the same blood sample showed that the white blood cell count was 22,740/μL, neutrophil count 22,060/μL, lymphocyte count 280/μL, LDH 654 U/L, creatinine kinase 22 U/L, CRP 3.89 mg/dL, and βD-glucan test was negative. Unfortunately, the patient eventually died of respiratory and hepatic failure 56 days after starting the steroid therapy.

## 3 Discussion

The patient died of drug-induced interstitial lung disease (DILD) that developed shortly after irinotecan-containing chemotherapy for advanced colorectal cancer more than 30 years after allogeneic BMT. Two mechanisms of DILD have been reported: 1) direct injury of lung epithelial cells and/or endothelial cells in lung capillaries by the drug and/or its metabolites (9) and 2) hypersensitivity reactions (10). Although immune reactions such as cytokines and T cell activation are involved, and BMT may alter the immune status of the host, the association between BMT and DILD is not well known.

Past and present lung disease and cumulative lung damage due to exposure to toxic agents such as smoking and radiation are well-known risk factors for DILD, and BMT also causes cumulative lung damage as complications such as GVHD (11). In addition to acute GVHD, which is one of the most common non-infectious complications after BMT (12), long-term pulmonary injury after BMT is known as chronic GVHD. Lung-related late toxicity after allogeneic stem cell transplantation is called a late-onset non-infectious pulmonary complication (LONIPC) (13–15). Lung function in patients with LONIPC declines over time after hematopoietic stem cell transplantation, and this decline has a significant impact on survival (16). Lung damage after BMT presents in various nonspecific forms (17), and bronchiolitis obliterans (BO) is a well-known pulmonary complication of chronic GVHD. Interstitial lung disease (ILD) has recently attracted attention as a pulmonary complication of chronic GVHD (18, 19). A large French retrospective observational cohort study including 79 ILD patients and 159 BO patients diagnosed after allogeneic BMT reported that 56% of ILD patients had experienced acute GVHD and 75% had developed chronic GVHD and that significantly fewer ILD patients had a history of steroid treatment for chronic GVHD compared to BO patients (62% vs 86%, *p* < 0.0001) (20). However, this large cohort study did not specifically address DILD. In the medical history of the present case, grade 1 acute GVHD, and non-symptomatic chronic GVHD without steroid treatment after BMT were consistent with the French retrospective study. Because no structural changes, such as fibrosis, were evident on initial lung imaging in the present case and his symptoms (cough) were judged to be caused by lung metastasis, it was difficult to recognize that he had risk factors for DILD other than a history of BMT before initiating chemotherapy.

Irinotecan has been associated with the risk of drug-induced lung injury in multiple case reports (21, 22). Lung and colorectal cancer are the two most common solid tumors in which irinotecan-related lung injury occurs (23), but its incidence is so low that it was not reported in large phase III clinical trials of irinotecan-containing chemotherapy for advanced colorectal cancer (24, 25). Similarly, in a clinical trial limited to the Japanese population, ILD incidence in patients receiving irinotecan-based and oxaliplatin-based chemotherapy were less than 1% (26). However, post-marketing surveillance has reported that the mortality rate of severe ILD after irinotecan administration was >20%. Therefore, it is important to monitor ILDs during chemotherapy with irinotecan (27). Although triplet chemotherapy, 5-fluorouracil/leucovorin + oxaliplatin + irinotecan (FOLFOXIRI) with bevacizumab, is an option for *BRAF*-mutated advanced colon cancer, a meta-analysis did not show the significant superiority of triplet chemotherapy (28). Another treatment option for *BRAF*-mutated colorectal cancer is doublet chemotherapy with a BRAF inhibitor and cetuximab (anti-epidermal growth factor receptor antibody). However, this doublet chemotherapy has no indication as first-line chemotherapy in Japan, and cetuximab treatment carries a risk of interstitial pneumonitis similarly to irinotecan and oxaliplatin. Doublet chemotherapy plus bevacizumab was considered optimal in the present case with a history of BMT. FOLFIRI and FOLFOX (5-fluorouracil/leucovorin + oxaliplatin) in combination with bevacizumab showed equivalent efficacy and safety, including for ILD (26). FOLFIRI plus bevacizumab was preferred in the present case after explaining the use of both FOLFIRI and FOLFOX.

Considering that ILD is a well-known adverse event caused by immune checkpoint inhibitors, another possible risk factor for DILD may be changes in pulmonary immunity caused by BMT. Mature T cells in the graft encounter and respond not only to tumor-associated and tumor-specific antigens, but also to host alloantigens, such as incompatible large histocompatibility leukocyte antigens, which cause GVHD, as well as graft-versus-tumor effects. Important advances have been made in our understanding of the role of regulatory T cells (Tregs) in immunomodulation; Tregs are involved in T cell self-regulation and to preferentially suppress alloreactive T cells (29). and act in a suppressive manner against tumor immunity (30). The T cell repertoire generated by thymus-independent mechanisms lacks diversity and is therefore biased. In the present case, it is possible that the immune editing mechanisms altered by BMT caused organ-specific autoimmune reactions (31). In the present case, the cause of DILD could not be identified because no specific findings were obtained in the BAL and in the biopsy by bronchoscopy. Moreover, because there were no apparent clinical manifestations of chronic GVHD, such as oral ulcers, keratoconjunctivitis, multiple sclerosis, esophagitis/stenosis, vaginal ulceration/stenosis, fasciitis, and myositis, it is unclear whether GVHD more than 30 years after BMT might be related to the respiratory failure. However, it cannot be ruled out that immunological changes, which could be a background factor activated by irinotecan, might finally cause the DILD. Future studies are warranted to clarify the immunological mechanism of DILD, and it would be meaningful to perform bronchoscopy for patients who develop DILD repeatedly, which can provide samples for translational research on the immunological environment.

Drug management during high-dose steroid therapy is important. Interstitial pneumonia encompasses a variety of diseases, and its clinical course and response to treatment differ depending on the cause and histopathological patterns. Idiopathic interstitial pneumonia, including idiopathic pulmonary fibrosis, is diagnosed when all identifiable pathogens are ruled out. In this case, the ground-glass shadows occurred shortly after starting chemotherapy and there was no obvious pathogen or exposure history; therefore, drug-induced lung injury was the most likely cause. The main therapeutic strategy includes discontinuation of the culprit drug and the use of steroid treatment for varying durations, guided by the clinical response. The reported efficacy of steroid treatment in DILD varied widely, the case of DILD accompanied by malignancies is often refractory to steroid therapy. Although there is no consensus or standard guidelines for the diagnosis and optimal treatment of DILD in cancer patients, administration of immunosuppressive agents, including biologic agents from an earlier stage is also suggested for treatment of steroid resistant DILD (32). In a study of 75 cancer patients with irinotecan-induced DILD treated with steroid treatment, over 60% of the patients recovered and 29% died (27). Other reports of drug-induced lung injury suggest that 50–100% of patients recover after drug discontinuation and steroid administration (10). Diffuse alveolar damage (DAD) patterns are less responsive to steroids and have a poor prognosis. In one study, none of the patients with DAD improved without steroid treatment, and the overall mortality rate was 37.5% (33). In the present case, the DAD pattern was not initially observed by chest CT or lung biopsy, and DILD improved rapidly after starting steroid therapy.

However, DILD flares in association with fibrosis and bronchiectasis after an episode of abdominal bleeding. The autoimmune disease field determined that steroid coverage should be considered for invasive interventions such as surgery in patients receiving prednisolone doses of 5 mg/kg/day or more for three weeks or longer (34). In retrospection, since the present case received arterial embolization for retroperitoneal hematoma and anterior superior pancreatic duodenal artery pseudoaneurysm after steroid use for more than three weeks, steroid coverage with the addition of hydrocortisone 100–150 mg/body/day or methylprednisolone 20–30 mg/body/day could have been administered.

CMV infection associated with liver dysfunction was detected near the end of the clinical course, and CMV-induced pneumonitis could not be ruled out. It has also been argued that patients receiving systemic steroids are more likely to require pre-emptive CMV treatment (35). Although, in this case, PCR for CMV DNA at the start of steroid treatment was negative and there were no pathologically positive cells, CMV antigenemia or PCR monitoring should be repeated, and preemptive CMV treatment should be considered during the long-term use of high-dose steroids.

Long-term survivors of BMT are at risk for secondary cancer, which might require specific treatment and care. In practice, however, treatment for patients who develop secondary cancers after BMT are scattered over long follow-up periods, resulting in few reports on chemotherapy for secondary cancers of BMT recipients. Therefore, large, retrospective, cohort and multicenter, prospective, observational studies are necessary to clarify whether BMT is a risk factor for DILD and other specific adverse events after chemotherapy.

## 4 Conclusion

This case suggests that BMT may be a risk factor for DILD.

## 5 Patient perspective

Even if a patient has had no specific problems in the long course of life after bone marrow transplantation, it is necessary to consider the possibility of suffering pulmonary damage and immune adverse events when suffering from a malignancy and receiving treatment.

## Data availability statement

The raw data supporting the conclusions of this article will be made available by the authors, without undue reservation.

## Ethics Statement

Written informed consent was obtained from the patient's family for the publication of this case report.

## Author contributions

KB and NB contributed substantially to the conception or design of the work or to the acquisition, analysis, and interpretation of data for the work. All authors agree to be responsible for all aspects of the work in ensuring that any questions relating to the accuracy or completeness of any part of the work are properly investigated and resolved. All authors contributed to the article and approved the submitted version.

## Supplementary material

Supplementary Figure 1 Hematoxylin and eosin staining of transbronchial lung biopsy of the right lung. Fibrosis is not evident on the left side, and fibrosis associated with cancer and carcinoma is present from the middle to the right, representing fibrotic changes in the tumor environment.

Supplementary Figure 2 The clinical course after onset of DILD.
