# Supplementary figures and images for: Case report: Irinotecan-induced interstitial lung disease in an advanced colorectal cancer patient resurfacing decades after allogeneic bone marrow transplantation for aplastic anemia; a case report and narrative review of literature
Source: Front Oncol. 2023 Jun 16;13:1215789. doi: 10.3389/fonc.2023.1215789 (PMC10313190; doi:10.3389/fonc.2023.1215789)

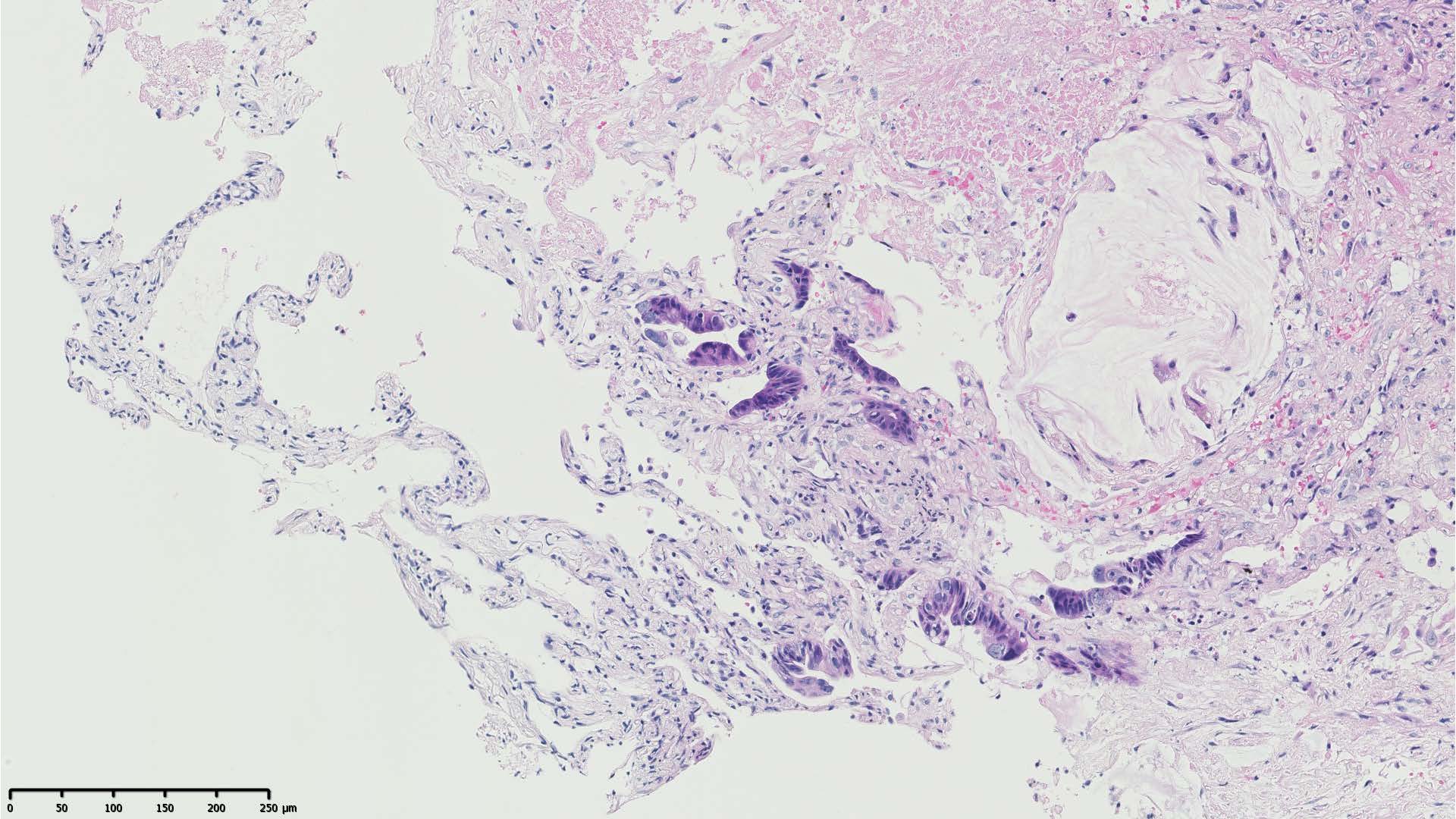

Supplement: Supplementary Figure 1 — Hematoxylin and eosin staining of transbronchial lung biopsy of the right lung. Fibrosis is not evident on the left side, and fibrosis associated with cancer and carcinoma is present from the middle to the right, representing fibrotic changes in the tumor environment. [file Image_1.jpeg]

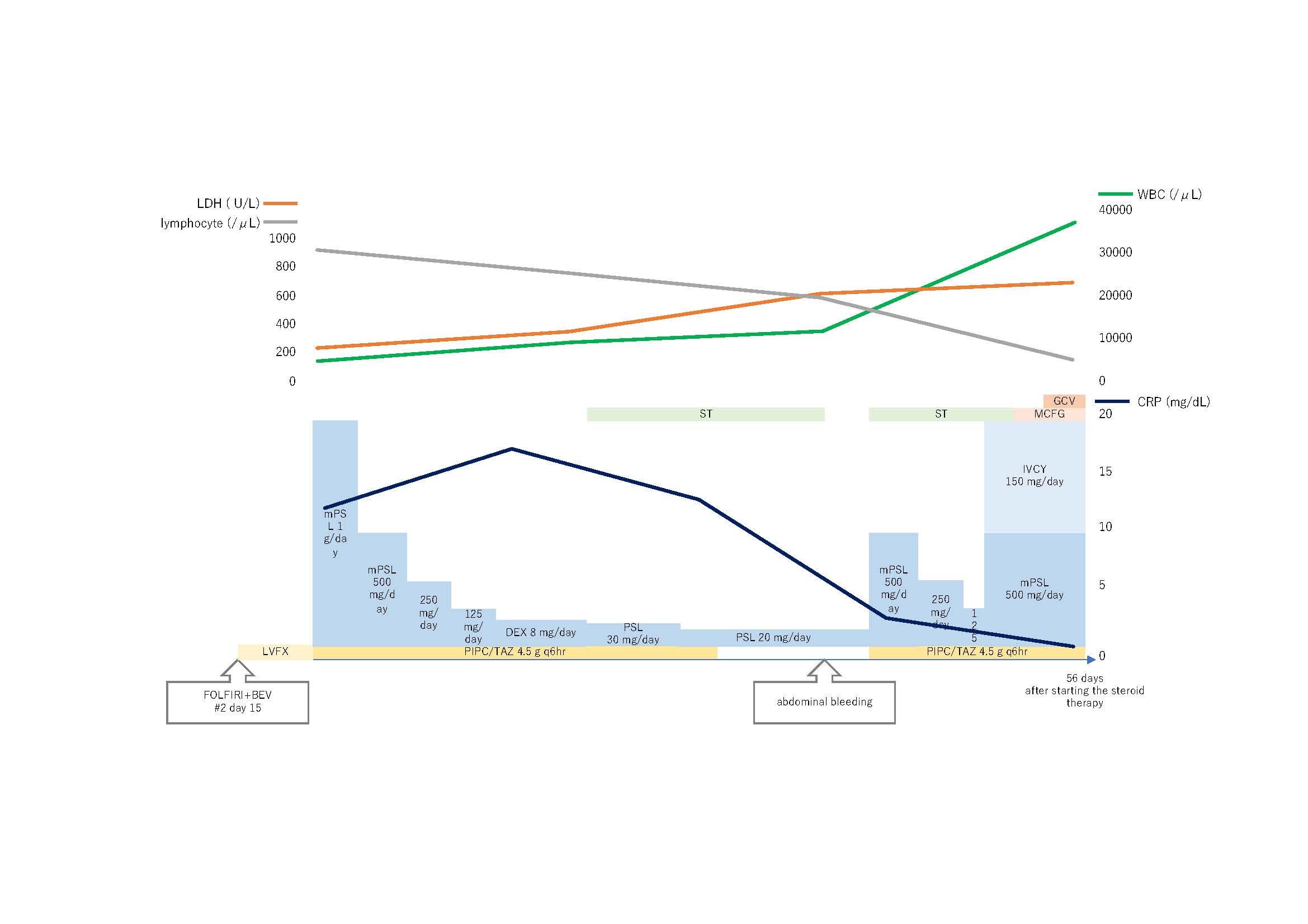

Supplement: Supplementary Figure 2 — The clinical course after onset of DILD. [file Image_2.jpeg]
